# Flavonoids from *Nelumbo nucifera* Gaertn., a Medicinal Plant: Uses in Traditional Medicine, Phytochemistry and Pharmacological Activities

**DOI:** 10.3390/medicines5040127

**Published:** 2018-11-23

**Authors:** Duangjai Tungmunnithum, Darawan Pinthong, Christophe Hano

**Affiliations:** 1 Department of Pharmaceutical Botany, Faculty of Pharmacy, Mahidol University, Bangkok 10400, Thailand; 2 Department of Pharmacology, Faculty of Science, Mahidol University, Bangkok 10400, Thailand; Darawan.pin@mahidol.ac.th; 3 Laboratoire de Biologie des Ligneux et des Grandes Cultures (LBLGC EA1207), INRA USC1328, Plant Lignans Team, Université d’Orléans, Pôle Universitaire d’Eure et Loir, 21 rue de Loigny la Bataille, 28000 Chartres, France; hano@univ-orleans.fr; 4 Bioactifs et Cosmétiques, GDR 3711 COSMACTIFS, CNRS/Université d’Orléans, 45067 Orléans CÉDEX 2, France

**Keywords:** *Nelumbo nucifera*, flavonoids, traditional medicine, pharmacological activities

## Abstract

*Nelumbo nucifera* Gaertn. has been used as an important ingredient for traditional medicines since ancient times, especially in Asian countries. Nowadays, many new or unknown phytochemical compounds from *N. nucifera* are still being discovered. Most of the current research about pharmacological activity focus on nuciferine, many other alkaloids, phenolic compounds, etc. However, there is no current review emphasizing on flavonoids, which is one of the potent secondary metabolites of this species and its pharmacological activities. Therefore, following a taxonomic description, we aim to illustrate and update the diversity of flavonoid phytochemical compounds from *N. nucifera*, the comparative analysis of flavonoid compositions and contents in various organs. The uses of this species in traditional medicine and the main pharmacological activities such as antioxidant, anti-inflammatory, anti-diabetic, anti-obesity, anti-angiogenic and anti-cancer activities are also illustrated in this works.

## 1. Introduction

**Figure 1:**
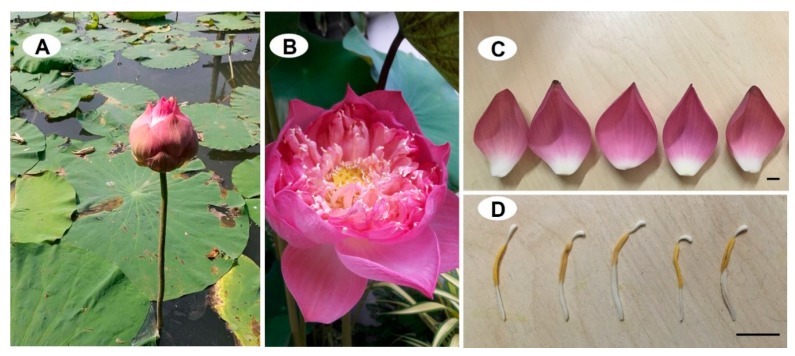
*Nelumbo nucifera* Gaertn.: (**A**) natural habitat; (**B**) flower; (**C**) perianth; and (**D**) dtamen. The photos were taken by D.T. Bar scale = 1 cm.

*Nelumbo nucifera* Gaertn. is an aquatic flowering plant belonging to the family nelumbonaceae. This perennial plant is well-known as various common names, e.g., sacred lotus, Indian lotus, Water lily and Chinese water lily. This species can be found mainly in Asian countries such as Thailand, China, Sri Lanka, India, Nepal, New Guinea or Japan [1,2,3]. Furthermore, *N. nucifera* is also distributed in Australia and Russia and was introduced to Western Europe and America long ago [4]. This plant is widely recognized by the beauty of its flowers, and has been considered a spiritual symbol for Buddhists, Hindus and Egyptians since ancient times. The flowers are very large and showy and considered sacred by Hindus, whereas the whole plant is holy according to Buddhists (Figure 1). Because of the beauty of its flower, it is also the national flower of India and Vietnam. Besides these considerations, there are also many advantages provided by this plant species such as being an ingredient for preparing various cuisines (such as its edible perianth, rhizomes and seeds) and also the important component for traditional medicines or herbal drugs [2,5,6,7,8,9], this latter point being the subject for many studies on phytochemical characterization and/or their bioactivities on this medicinal species, especially the leaves [1,2,3,4,5,6,7,8,9,10,11,12,13,14,15,16,17,18,19,20,21,22,23,24,25,26,27,28,29,30,31,32,33,34,35,36,37,38,39,40,41,42,43,44]. The present work highlights and updates on the comparative analysis of flavonoids and compositions in different organs of *N. nucifera* with an overview of main pharmacological activity from its flavonoid compounds. A proposed biosynthetic sequence of flavonoid *O*-glycosides (FOGs) as well as flavonoids *C*-glycosides (FCGs) derived flavonoids from this plant is also discussed. In addition, the taxonomic description and the uses in traditional medicine are also illustrated. 

## 2. Taxonomic Description of *Nelumbo nucifera* Gaertn

Aquatic perennial, rhizomatous. Petiole 2–1.3 m, terete, glabrous or papillae hard and scattered. Leaf orbicular, blue-green, 28.5–90.5 cm in diam., glabrous, glaucous, water-repellent, margin entire. Flowers 9.52–4.5 cm in diam. Peduncles longer than petioles, glabrous or sparsely spinulate. Perianth caducous, oblong, oblong-elliptic or obovate, pink or white, 4.5–10.5 × 2.7–5.5 cm. Stamens longer than receptacle, filament slender; anther linear, 2–1.5 mm; connective appendage. Receptacle accrescent, turbinate, 4.5–9.5 cm in diam. Ovary Superior. Fruit oblong or ovoid, 3.2–5 × 6.5–15 cm, glabrous, pericarp thick, hardened. Many herbarium specimens both from the past and current collection have been cross-checked in the major herbaria of the country for species authentication. 

## 3. *N. nucifera* is Used in Various Traditional Medicine

**Figure 2:**
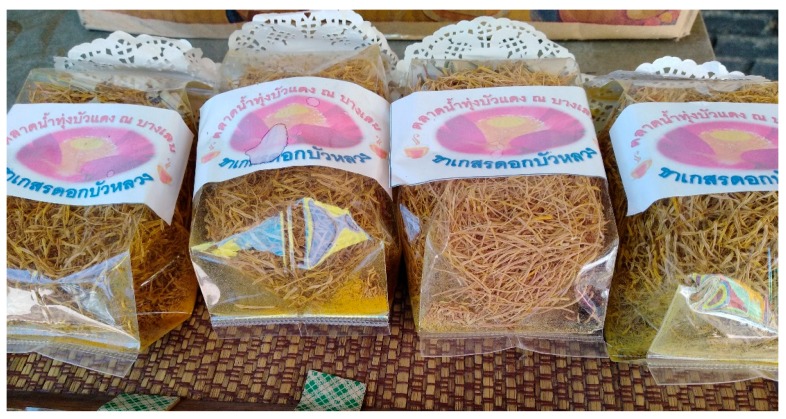
The herbal tea product from stamens of *Nelumbo nucifera* Gaertn. are sold in local markets. The photo was taken by D.T.

It is commonly known that *N. nucifera* or the lotus plant has been widely used as a component of traditional Chinese, Indian, Japanese, Thai, and Korean medicines and many others for several medicinal purposes [5,6]. The whole plant is used as an herbal medicine to cure diarrhea, insomnia, fever, body heat imbalance and gastritis [2,5,6,8]. In Korea, India and China, it is also used as a hemostatic [8]. Dry leaves and perianth of *N. nucifera* are consumed as health promoting teas. Yeon Yip Bap, a local food of Korea, also uses the leaves of *N. nucifera* as an ingredient [36]. Every part of this aquatic plant species—such as stamens, leaves, petioles, flowers, seeds and rhizomes—have been used for more than 1000 years in Chinese traditional medicines, and nowadays the production of *N. nucifera* leaves for traditional medicine usage and pharmaceutical industries is over 800,000 t/year in China [6,28]. The flowers and pedicels are used as both cardiac and hepatic tonics. The seeds are prepared to treat cutaneous diseases. They are also freshly eaten. The rhizome powder is used medicinally to promote the health balance (e.g., having positive effects on circulatory system). In China, leaves are used to fight against hyperlipidemia, hematemesis, metrorrhagia, fever treatment or to release skin inflammatory symptoms [6,8,28]. In addition, Asian people, practically in China and Taiwan, also prepare herbal tea from dry leaves of *N. nucifera* to lose weight and decrease body fat index [5,40]. In Thailand, the stamens constitute the necessary ingredient for preparing Thai traditional medicines. The herbal teas from stamens of *N. nucifera* are traditionally used by Thai people to improve the circulatory system, decrease blood glucose and blood lipid levels, and reduce oxidative stress substances in the body (Figure 2). 

## 4. Flavonoids from *N. nucifera* and Their Pharmacological Activities 

### 4.1. Phytochemical Diversity of *N. nucifera* Flavonoids

**Figure 3:**
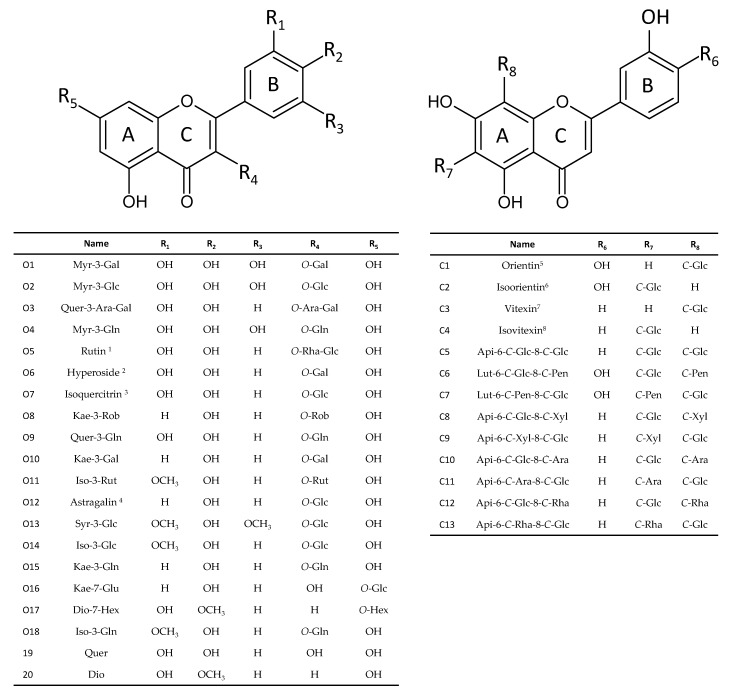
The chemical structures and names of the mains flavonoid *O*- (starting by O), *C*-glycosides (starting by C) and quercetin (Quer) and Diosmetin (Dio) aglycones from *Nelumbo nucifera* tissues. Flavonoid abbreviations used: Myr, myrycetin; Quer, quercetin; Kae, kaempferol; Iso, isorhamnetin; Syr, syringetin; Dio, diosmetin; Api, apigenin; Lut, luteolin. Sugar abbreviations used: Glc, glucoside; Gal, galactoside; Ara, arabionoside; Gln, glucuronide; Rut, rutinoside; Hex, hexoside; Xyl, xylose; Rha, rhamnose; Pen, pentose. ^1^ rutin, quercetin 3-*O*-rhamnopyranosyl-(1→6)-glucopyranoside; ^2^ hyperoside, quercetin 3-*O*-galactoside; ^3^ isoquercitrin, quercetin 3-*O*-glucoside; ^4^ Astragalin, kaempferol 3-*O*-glucoside; ^5^ orientin, luteolin 8-*C*-*β*-D-glucopyranoside; ^6^ isoorientin, luteolin 6-*C*-*β*-D-glucopyranoside; ^7^ vitexin, apigenin 8-*C*-*β*-D-glucopyranoside; ^8^ isovitexin, apigenin 6-*C*-*β*-D-glucopyranoside. Adapted from Chen et al. [25] and Li et al. [35].

From a chemical point of view, flavonoids are phenylpropanoids with C6-C3-C6 backbone consisting in two phenyl rings (rings A and B) associated with one heterocyclic ring (ring C) (Figure 3). In plants, flavonoids are generally accumulated in the forms of glycosides. Glycosylation step results in changes in solubility, stability and/or toxic potential of these compounds but can also influence their cellular compartmentalization and biological activities [45]. In nature, flavonoids can be found in two distinct *O*- and *C*-glycoside forms with different actions both in plant and in human health [46]. The *O*-glycosylation of flavonoids leading to the production of flavonoid *O*-glycosides (FOGs), which widely occurs in the plant kingdom, has been well characterized and described [23]. On the contrary, flavonoid C-glycosides (FCGs) production has received much less attention and the *C*-glycosyltransferase enzymes catalyzing this step are far less studied than their *O*-glycosyltransferase cousins [11]. By definition, contrary to *O*-glycosylation that consists in the transfer of the sugar moieties to the oxygen atom of a hydroxyl group of the flavonoid, *C*-glycosylation leads to the creation of a very stable carbon–carbon bond between the sugar moieties and the carbon skeleton of the flavonoid. This less common glyosidic bound is much more resistant to hydrolysis occurring under acidic pH or from enzyme action, and is supposed to lead to important changes in the biological roles and activities of the resulting FCGs as compared to their FOG counterparts. From a pharmacological point of view, this specific glycosylation step drastically changes the disponibility, pharmacokinetics and biological activities of the flavonoids, including their antioxidant, anti-inflammatory, hepatoprotective, antiviral and anticancer actions [11,32]. Lotus is one of the richest sources of a wide variety of flavonoids mainly accumulated in the forms of FOGs and FCGs. The main FOGs and FCGs accumulated in lotus tissues are presented in Figure 3. Zhu et al. [27] reported on a higher antioxidant potential of FCGs than FOGs from distinct lotus extracts. However, the effect of *C*-glycosylation on biological activities of the resulting flavonoids have been scarcely investigated in lotus. 

**Figure 4:**
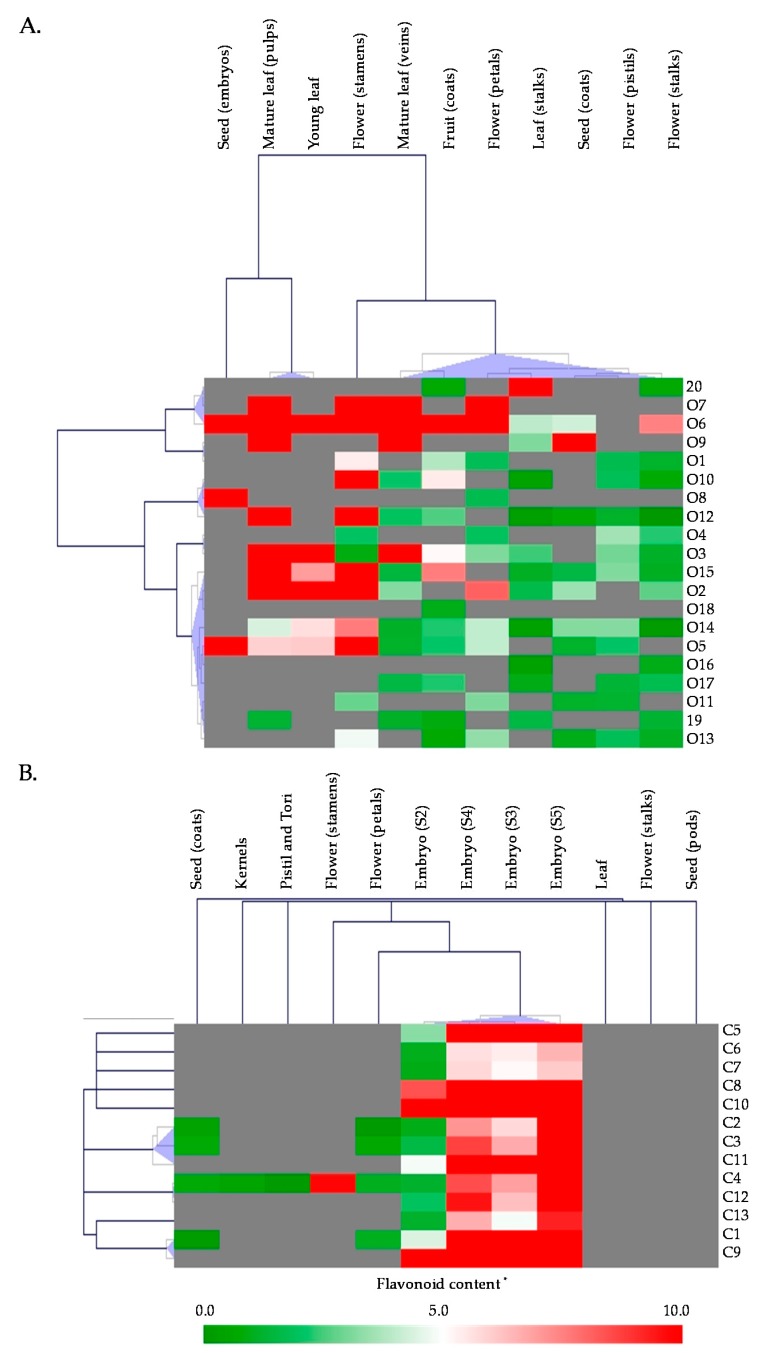
Comparative analysis of flavonoid contents and compositions different tissues of *Nelumbo nucifera*. Data were compiled from Chen et al. [33] and Li et al. [35]. (**A**) Comparative analysis of flavonoids aglycones and FOGs in 11 different tissues. (**B**) Comparative analysis of FCGs in 12 tissues and/or developmental stages including embryo (the major accumulation site of FCGs in *Nelumbo nucifera*) at five maturation stages. The names and structures of the flavonoids are presented in Figure 3. * Contents are expressed in mg.100 g^−1^ FW for all tissues except for seed embryo for which contents are expressed in mg.100 g^−1^ DW in (**A**)).

Interestingly, in lotus, FOGs and FCGs present a different accumulation pattern with a preferential accumulation of FCGs in embryo (in which they represent more than 70% of the total flavonoid content), whereas leaves also accumulate high levels of flavonoids but exclusively FOGs [5,27,33] (Figure 4). 

FOGs deriving from Quer **19** (hyperoside **O6**, isoquercitrin **O7** and Quer-3-Gln **O9**), Kae (astragalin **O12**) and Iso (Iso-3-Glc **O14**) were detected in all analyzed tissue with the exception of the embryo. A clear distinction between FOGs accumulation pattern in vegetative vs. reproductive tissues is observed. In leaves, FOGs derived from six different aglycones (Quer **19**, Kae, Iso, Lut, Dio **20** and Syr by order of abundance) with the Quer **19** derivatives rutin **O5**, hyperoside **O6** and isoquercitrin **O7** as main constituents [25,27,35]. In vegetative tissues, FOGs are accumulated at high levels, in particular in leaves tissues including young leaves, mature leaf pulps and mature leaf veins, in which the three Quer **19** derivatives hyperoside **O6**, Isoquercitrin **O7** and Quer-3-Gln **O9** account for more than 70% of the total flavonoid content [35]. We can note that the three Kae derivatives were dominant in floral tissues petals and stamens accounting for more than 60% of the total flavonoid content, indicating large differences in the biosynthesis and accumulation in these tissues that accumulate the lowest contents of flavonoids. FOGs deriving from Myr (Myr-3-Gal **O1**, Myr-3-Gln **O4**), Kae (Kae-3-Rob **O8**), Iso (Iso-3-Rut **O11**) and Syr (Syr-3-Glc **O13**) were detected in all reproductive tissues but not vegetative tissues. Quantitatively, we can note that, with the exception of seed coat (not surprisingly, considering the maternal origin of this tissue), all reproductive tissue accumulated far less FOGs than the vegetative tissues. Particularly, FOGs were far less represented in embryo than in other lotus tissue and were even almost undetected in seed kernels. However, embryo accumulated high levels of FCGs [25,27,35]. The main FCGs accumulated in embryo derived from Api and to a less extend from Lut, with the sole Api-6-*C*-Glc-8-*C*-Ara **C10** (schaftoside) accounting for more than 35% of the total flavonoids content in this tissue (Figure 4) [35]. Note that this very specific FCG accumulation in embryo might have taxonomic implications for possible authentication based on the association of these compounds as possible chemotaxonomic markers [47]. 

Besides these tissue-specific accumulations of FOGs vs. FCGs, the flavonoid composition and contents in lotus is also dependent of plant development. During development, an increase is observed in leaves, petals, stamens, pistils and tori for FOGs and embryo for both FOGs and FCGs. On the contrary, FOGs decreased during development in flower stalks, seed coats and kernels, whereas it remained constant in seed pods. In leaves, Quer-3-Gln **O9** is the major contributor of this variation during development. In embryo, the composition and content are extremely different with a continuous increase of Api-derived FCGs. We can also note that the variation observed in petals is much more complex with a global decrease during development but a very complex accumulation kinetics of the different constituents that need further studies [35]. 

The composition and contents of flavonoids also greatly vary according to the genetic background. Indeed, Chen et al. [25] reported on the differences in three lotus cultivars (Honglian, Baijian and Zhimahuoulian). Quer-3-Glc was the dominant FOG detected in the leaves of these cultivars but quantitative variations were observed. Honglian accumulated 1.8 times more isoquercitrin **O7** than Baijian, whereas this latter cultivar appeared as the richest source of flavonoids compared to the two other cultivars including Honglian. This could be explain by the fact that, contrary to the two other cultivars, Kae-3-*O*-Gal was absent in Honglian [25]. These observations could lead to the potential use of some flavonoids in authentication of *N. nucifera* cultivars for specific medicinal applications based on their flavonoid accumulation profiles [47]. 

**Figure 5:**
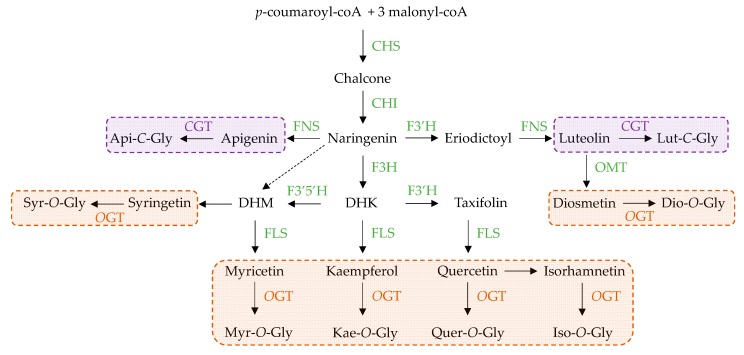
Proposed biosynthetic sequence of FOGs (orange) and FCGs (purple) derived flavonoids from *Nelumbo nucifera* adapted from Li et al. (2014). CHS, chalcone synthase; CHI, chalcone isomerase; FNS, flavone synthase; F3H, flavanone 3 hydroxylase; F3′H, flavonoid 3′ hydroxylase; F3′5′H, flavonoid 3′,5′ hydroxylase; FLS, flavonol synthase; *O*GT, *O*-flavonoid glycosyltransferase; CGT, *C*-flavonoid glycosyltransferase; Api, apigenin; DHM, dihydromyricetin; DHK, dihydrokaempferol; Myr, myricetin; Kae, kaempferol; Quer, quercetin; Iso, isorhamnetin; Dio, diosmetin; Lut, luteolin; Syr, syringetin; Gly, glycoside(s).

Flavonoid biosynthetic pathway starts with the condensation of one *p*-coumaroyl-coA together with 3 malonyl-coA moieties catalyzed by chalcone synthase (CHS) leading to the synthesis of chalcone, the first committed flavonoid (Figure 5). Various hydroxylation, methylation and acetylation steps follow this first condensation and lead to a wide variety of derivatives including the flavonols and flavones accumulating in the louts tissues: Api, Dio **20**, Iso, Kae, Lut, Myr, Quer **19** and Syr. The aglycones could then be glycolsylated at different positions by a wide of glycosyltransferases branching a wide range of sugar moieties resulting in the great variety of FOGs and FCGs observed in lotus tissues. In lotus, two types of glycosyltransferases act with a very distinct distribution pattern [35]. Considering the FOGs distribution *O*-flavonoid glycosyltransferases must be highly active in leaves and much more ubiquitous than the *C*-flavonoid glycosyltransferases that are probably restricted to the embryo according to the high FCGs accumulation almost circumscribed to this tissue. These two tissues (leaves vs. embryo) constitute attractive starting materials to isolate these distinct classes of flavonoid glycosyltransferases from lotus for further characterization. Interestingly, from a structural point of view FCGs accumulated in embryo derived from Api and to a less extent from Lut, whereas FOGS accumulated in leaves derived from Quer **19** and to a less extent from Kae, Iso, Lut, Dio **20** and Syr. A regulation occurring at the level of the F3H enzyme (with the possible exception of Dio **20** if its biosynthetic sequence is conserved in lotus) could be responsible for these specific structural accumulation patterns (Figure 5). Once again, we can anticipate that the contrasting accumulation profiles of leaves vs. embryo could be used to elucidate these regulation flavonoid biosynthetic pathways at both molecular and biochemical levels to make the most potential of these compounds. We anticipate that future experiments using labeled compounds to elucidate the biosynthetic sequence of the different flavonoids and/or RNA sequencing technologies could be very informative.

### 4.2. Antioxidant Activities

It is well accepted that many degenerative diseases are the consequence of oxidative stress and caused by reactive oxygen species (ROS) and reactive nitrogen species (RNS). These ROS and RNS can damage many important organelles and molecules such as DNA, lipids and protein in the cells [6,7,48]. Jung et al. reported on the antioxidant potential from stamen methanolic extract of *N. nucifera* plant collected from Korea, investigating the main phytochemical compounds from this extract [8]. They evidenced the flavonoid Kae as the main contributor of the antioxidant potential of these extracts. For that, they used both in vitro DPPH assay and in vivo animal model, showing the inhibition of ROS generation from kidney homogenates of Wistar rats kidneys by using the 2′,7′-dichlorodihydrofluorescein diacetate (DCHF-DA) probe [8]. This observation is in good agreement with the study of Rai et al. investigating both in vitro and in vivo antioxidant activity of a flavonoid-rich 50% (*v*/*v*) hydroalcoholic extract from *N. nucifera* seeds [7]. The absence of acute toxicity even at the highest oral administration dose of 1000 mg/kg body weight using Swiss Albino mice in vivo model of these extracts have also been described [6,7,48]. Besides, administration to Wistar rats in vivo model prior to carbon tetrachloride treatment produced a significant dose dependent increase in the level of superoxide dismutase, key enzyme scavenging the superoxide radicals [7]. Catechin, Quer **19**, Isoquercitrin **O7**, Quer-3-Gln **O9**, hyperoside **O6**, astragalin **O12** and Myr-3-Glc **O2** were isolated from the leaves extract of *N. nucifera* from Taiwan by Lin et al. [9]. They evidenced the first four as potent inhibitors of LDL oxidation, whereas Myr-3-Glc **O2** showed the more pronounced DPPH scavenging activity. Consequently, these results confirmed that the antioxidant activity of lotus leaves extract relied on both their flavonoid content and composition [9]. Interestingly, Chen et al. [49] studied antioxidant potential of the seed epicarp of *N. nucifera*, a flavonoid rich tissue considered as a byproduct, which displayed a strong in vitro antioxidant activity, as revealed by ABTS, DPPH and FRAP assays [49]. This byproduct could therefore be considered as a potential source for the functional food industry in the future. Furthermore, Liu et al. also examined the antioxidant activity from epicarp of *N. nucifera* seed in China at different ripening stages: green, half ripe and fully ripe stages [29]. They identified catechin, epicatechin, hyperoside **O6** and isoquercitrin **O7** at different levels depending on the ripening stages. The scavenging abilities revealed by the DPPH and ABTS in vitro assays were very effective but decreased during maturation [29]. Besides, Zhu et al. identified 14 flavonoids including four new compounds, Quer-3-*O*-Ara, Quer-3-*O*-Rha-(1→2)-Glc, Dio-7-Hex **O17**, and Iso-3-*O*-Ara-(1→2)-Glc, from their lotus leaves extract exhibiting a strong antioxidant potential [6]. Twenty flavonoids were identified in plumules extract of *N. nucifera* by Feng et al., and 14 flavonoids were under the form of FCGs [21]. In this study, the authors analyzed 38 different cultivars of lotus species, and evaluated their antioxidant activity by DPPH and FRAP in vitro assays. Their results clearly showed a significant positive correlation between the total polyphenol content and the antioxidant potential of the extract. Among these cultivars, four cultivars (Taikonglian, Yinqiu, Jinqi and Hongtailian) appeared as the most suitable cultivars for their use in healthcare products according to their strong antioxidant activity [21]. Additionally, eight FCGs and eight FOGs from embryos of *N. nucifera* were identified by Zhu et al. [27] and the FCGs were recognized as the major flavonoid forms found in these lotus seed embryo extract. Kae-7-Glc **O16** and luteolin 7-*O*-neohesperidoside were described for the first time in *N. nucifera* embryos. These authors also compared the antioxidant potential of extracts from seed embryos vs. leaves, and pointed that embryo extract exhibited at least a comparable or even higher antioxidant activity than leaves extracts. However, note that in lotus the total flavonoid content was lower in embryo than in leaves. Interestingly, these authors also pointed that the antioxidant potential of FCGs from *N. nucifera* embryo extracts was higher than that of FOGs from leaf extracts [26]. In addition, Jiang et al. identified four new FCGs (named nelumbosides A–D) from lotus embryo and evaluated their relative antioxidant activity using ABTS and DPPH assays [50]. They found that nelumbosides B exhibited the most promising radical scavenging activity [50].

### 4.3. Anti-Inflammatory Activities

Inflammation is part of a complex biological response of immune system to irritants, pathogens, damaged cells or other harmful stimuli. These complex processes deal with various biological pathways. Kim et al. investigated age-related effects of Kae, a flavonoid from *N. nucifera,* on ROS and GSH oxidative status in in vivo model [39]. Their results showed a reduction of ROS production and GSH level augmentation following Kae supply in a dose-dependent manner. They also evidenced a significant reduction in the levels of iNOS and TNF-α protein. The authors proposed that Kae may inhibit ROS generation by decreasing the gene expression of iNOS and TNF-α in aged gingival rat tissues via the NF-κB and mitogen-activated protein kinase (MAPK) signaling pathways. The authors also examined the hypothesis that Kae anti-inflammatory effects was mediated via glutathione and NF-κB levels modulation. Altogether, their results pointed out Kae as a potent anti-inflammatory compound [39]. The anti-inflammatory activity of Quer-3-Gln **O9** from *N. nucifera* leaves was also evaluated by Li et al. using lipopolysaccharide-treated RAW264.7 macrophages and showed that Quer-3-Gln **O9** inhibited LPS-induced NO release [40].

### 4.4. Anti-Diabetic and Anti-Obesity Activities

Aldose reductase (AR) is the key enzyme in polyol pathway and has been identified as a convenient drug target for type II diabetic treatment. Lim et al. showed that the flavonoids isolated from stamen methanolic extract from *N. nucifera* can inhibit rat lens AR activity with the highest inhibition degree observed for flavonoids with a 3-O-α-l-Rha-(1→6)-β-d-Glc substitution on their C rings (i.e., Kae-3-*O*-α-l-Rha-(1→6)-β-d-Glc and Iso-3-*O*-α-l-Rha-(1→6)-β-d-Glc) [22]. Ohkoshi et al. demonstrated that flavonoids extract from *N. nucifera* leaves stimulated lipolysis in white adipose tissue of in vivo mice model [38]. In addition, flavonoid-rich extracts from *N. nucifera* leaves was also able to regulate insulin secretion as well as blood glucose level [43] in both in vitro and in vivo models. The authors proposed a possible enhancement of the insulin secretion from β-cells through a Ca^2+^-activated PKC-regulated ERK1/2 signaling pathway. They pointed out catechin as an active compound from this extract able to enhance insulin secretion in a dose-dependent manner. Indeed, oral administration of catechin at 100 mg/kg in fasted normal mice model 2 h before starch loading showed that catechin administration resulted in hypoglycemic effect on fasted mice. Furthermore, the same oral dose of catechin was able to reverse glucose intolerance in high-fat-diet-induced diabetic animal model. These results obtained from both in vitro and animal models showed that both *N. nucifera* leaves extract and catechin regulated glucose blood level and could improve postprandial hyperglycemia under diabetic conditions. These results strongly suggested that *N. nucifera* leaves extract and catechin are of particular interest to control hyperglycemia in non-insulin dependent diabetes mellitus [43]. However, the exact mechanism for the action of catechin on β-cells and glucose metabolism have to be further investigated and confirmed. 

Obesity is considered as an important risk factor for many chronic diseases including type 2 diabetes. There are many attempts to find natural compounds that have anti-obesity abilities. Sergent et al. searched for pancreatic lipase inhibitors to prevent obesity, finding epigallocatechin-3-gallate, Kae and Quer **19** as effective pancreatic lipase natural inhibitors [18]. Ahn et al. also confirmed that flavonoid-rich *N. nucifera* leaves showed the anti-obesity potential to inhibit pancreatic lipase, but also adipocyte differentiation [35]. In the same direction, Liu et al. evaluated the inhibitory effects of total flavonoids *N. nucifera* leaves extract on α-glucosidase, α-amylase, pancreatic lipase and hypolipidemia activities [31]. Their results showed that a *N. nucifera* leaf extract containing flavonoids (mainly FOGs) could ameliorate hyperlipidemia by inhibiting these key enzymes related to the type 2 diabetes mellitus. You et al. proposed the rhizome extracts from *N. nucifera* as a nutraceutical component against obesity-related diseases, including diabetes mellitus thanks to their potent anti-adipogenic effect in human pre-adipocytes in vivo experiment and using rats fed with a high-fat diet [41]. Interestingly, Kae isolated from *N. nucifera* stamen inhibited lipogenic transcription factors and the accumulation of lipid through PPARα binding as well as the stimulation of fatty acid oxidation signaling in several adipocyte cells [12]. Besides, Sharma et al. also validated the traditional use of *N. nucifera* leaves for diabetes treatment and showed that *N. nucifera* leaf extract attenuated pancreatic β-cells toxicity induced by interleukin-1β and interferon-γ, and increased insulin secretion of pancreatic β-cells in streptozotocin-induced diabetic rats in vivo study [16]. Recently, Wang et al. demonstrated that *N. nucifera* leaf flavonoids could prevent diabetes type 2 through the inhibition of α-amylase [51]. In the same way, Liao et al. analyzed the binding affinity of ten flavonoids from *N. nucifera* leaf on α-amylase using spectroscopic methods [52]. They found that, among the tested flavonoids, Kae, Api and Iso displayed the most potent inhibiting potential on α-amylase activity. Their structure–function experiments also showed that the hydrogenation of the C_2_=C_3_ double bond of the flavonoid backbone of Quer and Api as well as the hydroxylation of 3 and 3′ positions decreased the affinity of the flavonoids for this enzyme [52]. Additionally, the methanol extract from seed epicarp of *N. nucifera* revealed the significant α-amylase inhibiting activity, and was suggested to develop as potential anti-diabetic agents [49].

### 4.5. Anti-Angiogenic and Anti-Cancer Activities

One important approach for cancer therapy is to inhibit the angiogenesis. Lee et al. were the first to report on the potential *N. nucifera* leaves extract to inhibit vascular endothelial growth factor-induced angiogenesis using both in vitro and in vivo models [5]. Yang et al. also evaluated the anti-cancer effect of the flavonoids from *N. nucifera* leaves extracts (i.e., mainly FOGs) using human MCF-7 cell line and in vivo study using a xenograft nude mouse model [44]. Their results evidenced the anti-proliferative action of the flavonoids from *N. nucifera* leaf extracts on of breast cancer in both in vitro and in vivo models [44]. In addition, Wu et al. investigated the potential of *N. nucifera* leaves extract on breast cancer metastasis using both in vitro MDA-MB-231 and 4T-1 breast cancer cells and in vivo mice model through PKCα targeting [17]. Their result illustrated the effectiveness of the extracts for the development of potential chemopreventive agents to reduce breast cancer metastasis. The authors also showed the capacity of *N. nucifera* leaves extracts to inhibit the angiogenesis and metastasis of this breast cancer cells by down regulating the connective tissue growth factor mediated by PI3K/AKT/ERK signaling pathway. However, each cancer type resulting from many factors such as genetic or epigenetic factors, the chemopreventive agents or anti-cancer molecules here evidenced may possibly play distinct roles and regulate more than a single pathway. Thus, the anti-cancer actions of flavonoids from *N. nucifera* leaves extracts need to be investigate more specifically with different cancer types to gain more complete information. 

## 5. Conclusions and Future Research Directions

*Nelumbo nucifera* Gaertn. is one of the most important medicinal plants used in various traditional medicines. *N. nucifera* is a natural source of many potent flavonoids exhibiting various effective pharmacological activities. Nowadays, many pharmacological activities such as anti-angiogenic, anti-cancer, anti-diabetic and anti-obesity activities of flavonoids isolated from this medicinal species have been described. However, most of these pharmacological activities still need further research investigations before leading to the discovery of potent drugs from the extracts of this plant species and their large-scale development. 

In particular, from this literature review, several points appeared essential for future perspectives and research directions:

(1) To reach the maximum potential of this species as the raw plant material and the source of potential flavonoids, the cultivars, developmental stages, parts of plant, seasons and time to harvest are the major factors that should be considered.
(2) In the use of traditional medicines, there are many local names of this species depending on the areas and country. Some other plant may also be called by a similar name. Therefore, species authentication needs to be done before using it for medical and pharmaceutical applications.
(3) The geographic regions of the raw material should also be considered and compared in the future research from various Asian countries to determine the effect of environmental factors on the quality and quantity of phytochemicals.
(4) As a consequence of the last point, not only local medicinal plant species but also the wild and/or various local ecotypes are interesting for the future studies to discover novel phytochemical compounds to increase the alternative sources of raw material for medical and pharmaceutical applications. Research on the profiling and identification of new flavonoids is still a future challenge for the many unknown reported areas of Asia.
(5) For many pharmacological activities, the molecular mechanisms and/or signaling pathways need to be further clarified in the future research. In particular, there are few works to date on the biological activities of purified flavonoid C-glycosides (FCGs) from lotus.
(6) The indications of losing activity or adverse effects following prolonged exposure of extract or products should be investigated in future research studies.

## References

[1] Chen S., Zheng Y., Fang J.B., Liu Y.L., Li S.H. (2013). Flavonoids in lotus (Nelumbo) leaves evaluated by HPLC-MSnat the germplasm level. Food Res. Int..

[2] Sheikh S.A. (2014). Ethno-medicinal uses and pharmacological activities of lotus (*Nelumbo nucifera*). J. Med. Plants Stud..

[3] Deng J., Chen S., Yin X., Wang K., Liu Y., Li S., Yang P. (2013). Systematic qualitative and quantitative assessment of anthocyanins, flavones and flavonols in the petals of 108 lotus (*Nelumbo nucifera*) cultivars. Food Chem..

[4] Paudel K.R., Panth N. (2015). Phytochemical profile and biological activity of *Nelumbo nucifera*. Evid.-Based Complement. Altern. Med..

[5] Lee J.S., Shukla S., Kim J.A., Kim M. (2015). Anti-angiogenic effect of *nelumbo nucifera* leaf extracts in human umbilical vein endothelial cells with antioxidant potential. PLoS ONE.

[6] Zhu M.Z., Wu W., Jiao L.L., Yang P.F., Guo M.Q. (2015). Analysis of flavonoids in lotus (*Nelumbo nucifera*) leaves and their antioxidant activity using macroporous resin chromatography coupled with LC-MS/MS and antioxidant biochemical assays. Molecules.

[7] Rai S., Wahile A., Mukherjee K., Saha B.P., Mukherjee P.K. (2006). Antioxidant activity of *Nelumbo nucifera* (sacred lotus) seeds. J. Ethnopharmacol..

[8] Jung H.A., Kim J.E., Chung H.Y., Choi J.S. (2003). Antioxidant principles of *Nelumbo nucifera* stamens. Arch. Pharm. Res..

[9] Lin H.Y., Kuo Y.H., Lin Y.L., Chiang W. (2009). Antioxidative effect and active components from leaves of lotus (*Nelumbo nucifera*). J. Agric. Food Chem..

[10] Courts F.L., Williamson G. (2015). The occurrence, fate and biological activities of c-glycosyl flavonoids in the human diet. Crit. Rev. Food Sci. Nutr..

[11] Lee B., Kwon M., Choi J.S., Jeong H.O., Chung H.Y., Kim H.-R. (2015). Kaempferol Isolated from *Nelumbo nucifera* Inhibits Lipid Accumulation and Increases Fatty Acid Oxidation Signaling in Adipocytes. J. Med. Food.

[12] Zhu Y.T., Jia Y.W., Liu Y.M., Liang J., Ding L.S., Liao X. (2014). Lipase ligands in *nelumbo nucifera* leaves and study of their binding mechanism. J. Agric. Food Chem..

[13] Bin X., Jin W., Wenqing W., Chunyang S., Xiaolong H., Jianguo F. (2011). *Nelumbo nucifera* alkaloid inhibits 3T3-L1 preadipocyte differentiation and improves high-fat diet-induced obesity and body fat accumulation in rats. J. Med. Plant. Res..

[14] Chang C.H., Ou T.T., Yang M.Y., Huang C.C., Wang C.J. (2016). *Nelumbo nucifera* Gaertn leaves extract inhibits the angiogenesis and metastasis of breast cancer cells by downregulation connective tissue growth factor (CTGF) mediated PI3K/AKT/ERK signaling. J. Ethnopharmacol..

[15] Sharma B.R., Kim M.S., Rhyu D.Y. (2016). *Nelumbo Nucifera* leaf extract attenuated pancreatic beta-cells toxicity induced by interleukin-1beta and interferon-gamma, and increased insulin secrection of pancreatic beta-cells in streptozotocin-induced diabetic rats. J. Tradit. Chin. Med..

[16] Wu C.H., Yang M.Y., Lee Y.J., Wang C.J. (2017). *Nelumbo nucifera* leaf polyphenol extract inhibits breast cancer cells metastasis in vitro and in vivo through PKCα targeting. J. Funct. Foods.

[17] Sergent T., Vanderstraeten J., Winand J., Beguin P., Schneider Y.J. (2012). Phenolic compounds and plant extracts as potential natural anti-obesity substances. Food Chem..

[18] Rajput M.A., Khan R.A. (2017). Phytochemical screening, acute toxicity, anxiolytic and antidepressant activities of the *Nelumbo nucifera* fruit. Metab. Brain Dis..

[19] Mongkolrat S., Palanuvej C., Ruangrungsi N. (2012). Quality assessment and liriodenine quantification of *Nelumbo nucifera* dried leaf in Thailand. Pharmacogn. J..

[20] Feng C.Y., Li S.S., Yin D.D., Zhang H.J., Tian D.K., Wu Q., Wang L.J., Su S., Wang L.S. (2016). Rapid determination of flavonoids in plumules of sacred lotus cultivars and assessment of their antioxidant activities. Ind. Crops Prod..

[21] Lim S.S., Jung Y.J., Hyun S.K., Lee Y.S., Choi J.S. (2006). Rat lens aldose reductase inhibitory constituents of *Nelumbo nucifera* stamens. Phyther. Res..

[22] Hofer B. (2016). Recent developments in the enzymatic O-glycosylation of flavonoids. Appl. Microbiol. Biotechnol..

[23] Wu H.M., Kao C.L., Huang S.C., Li W.J., Li H.T., Chen C.Y. (2017). Secondary Metabolites from the Stems of *Nelumbo nucifera* cv. Rosa-plena. Chem. Nat. Compd..

[24] Chen S., Fang L., Xi H., Guan L., Fang J., Liu Y., Wu B., Li S. (2012). Simultaneous qualitative assessment and quantitative analysis of flavonoids in various tissues of lotus (*Nelumbo nucifera*) using high performance liquid chromatography coupled with triple quad mass spectrometry. Anal. Chim. Acta.

[25] Zhu F. (2017). Structures, properties, and applications of lotus starches. Food Hydrocoll..

[26] Zhu M., Liu T., Zhang C., Guo M. (2017). Flavonoids of Lotus (*Nelumbo nucifera*) Seed Embryos and Their Antioxidant Potential. J. Food Sci..

[27] Huang B., Ban X., He J., Tong J., Tian J., Wang Y. (2010). Hepatoprotective and antioxidant activity of ethanolic extracts of edible lotus (*Nelumbo nucifera* Gaertn.) leaves. Food Chem..

[28] Liu Y., Ma S.S., Ibrahim S.A., Li E.H., Yang H., Huang W. (2015). Identification and antioxidant properties of polyphenols in lotus seed epicarp at different ripening stages. Food Chem..

[29] Huang B., Zhu L., Liu S., Li D., Chen Y., Ma B., Wang Y. (2013). In vitro and in vivo evaluation of inhibition activity of lotus (*Nelumbo nucifera* Gaertn.) leaves against ultraviolet B-induced phototoxicity. J. Photochem. Photobiol. B Biol..

[30] Liu S., Li D., Huang B., Chen Y., Lu X., Wang Y. (2013). Inhibition of pancreatic lipase, α-glucosidase, α-amylase, and hypolipidemic effects of the total flavonoids from *Nelumbo nucifera* leaves. J. Ethnopharmacol..

[31] Xiao J., Capanoglu E., Jassbi A.R., Miron A. (2016). Advance on the Flavonoid C-glycosides and Health Benefits. Crit. Rev. Food Sci. Nutr..

[32] Chen S., Wu B.H., Fang J.B., Liu Y.L., Zhang H.H., Fang L.C., Guan L., Li S.H. (2012). Analysis of flavonoids from lotus (*Nelumbo nucifera*) leaves using high performance liquid chromatography/photodiode array detector tandem electrospray ionization mass spectrometry and an extraction method optimized by orthogonal design. J. Chromatogr. A.

[33] Charbe N.B., McCarron P.A., Lane M.E., Tambuwala M.M. (2017). Application of three-dimensional printing for colon targeted drug delivery systems. Int. J. Pharm. Investig..

[34] Li S.S., Wu J., Chen L.G., Du H., Xu Y.J., Wang L.J., Zhang H.J., Zheng X.C., Wang L.S. (2014). Biogenesis of C-glycosyl flavones and profiling of flavonoid glycosides in lotus (*Nelumbo nucifera*). PLoS ONE.

[35] Ahn J.H., Kim E.S., Lee C., Kim S., Cho S.H., Hwang B.Y., Lee M.K. (2013). Chemical constituents from *Nelumbo nucifera* leaves and their anti-obesity effects. Bioorganic Med. Chem. Lett..

[36] Wang H.M., Yang W.L., Yang S.C., Chen C.Y. (2011). Chemical constituents from the leaves of *Nelumbo nucifera* gaertn. cv. Rosa-plena. Chem. Nat. Compd..

[37] Ohkoshi E., Miyazaki H., Shindo K., Watanabe H., Yoshida A., Yajima H. (2007). Constituents from the leaves of *Nelumbo nucifera* stimulate lipolysis in the white adipose tissue of mice. Planta Med..

[38] Kim H.K., Park H.R., Lee J.S., Chung T.S., Chung H.Y., Chung J. (2007). Down-regulation of iNOS and TNF-α expression by kaempferol via NF-κB inactivation in aged rat gingival tissues. Biogerontology.

[39] Li F., Sun X.Y., Li X.W., Yang T., Qi L.W. (2017). Enrichment and separation of quercetin-3-O-β-D-glucuronide from lotus leaves (*nelumbo nucifera* gaertn.) and evaluation of its anti-inflammatory effect. J. Chromatogr. B Anal. Technol. Biomed. Life Sci..

[40] You J.S., Lee Y.J., Kim K.S., Kim S.H., Chang K.J. (2014). Ethanol extract of lotus (*Nelumbo nucifera*) root exhibits an anti-adipogenic effect in human pre-adipocytes and anti-obesity and anti-oxidant effects in rats fed a high-fat diet. Nutr. Res..

[41] Ho H.H., Hsu L.S., Chan K.C., Chen H.M., Wu C.H., Wang C.J. (2010). Extract from the leaf of nucifera reduced the development of atherosclerosis via inhibition of vascular smooth muscle cell proliferation and migration. Food Chem. Toxicol..

[42] Huang C.F., Chen Y.W., Yang C.Y., Lin H.Y., Way T. Der, Chiang W., Liu S.H. (2011). Extract of lotus leaf (*Nelumbo nucifera*) and its active constituent catechin with insulin secretagogue activity. J. Agric. Food Chem..

[43] Yang M.Y., Chang Y.C., Chan K.C., Lee Y.J., Wang C.J. (2011). Flavonoid-enriched extracts from *Nelumbo nucifera* leaves inhibits proliferation of breast cancer in vitro and in vivo. Eur. J. Integr. Med..

[44] Ruvanthika P.N., Manikandan S., Lalitha S. (2017). A comparative study on phytochemical screening of aerial parts of *Nelumbo nucifera* Gaertn. by gas chromatographic mass spectrometry. Int. J. Pharm. Sci. Res..

[45] Le Roy J., Huss B., Creach A., Hawkins S., Neutelings G. (2016). Glycosylation Is a Major Regulator of Phenylpropanoid Availability and Biological Activity in Plants. Front. Plant Sci..

[46] Ferreres F., Gil-Izquierdo A., Andrade P.B., Valentão P., Tomás-Barberán F.A. (2007). Characterization of C-glycosyl flavones O-glycosylated by liquid chromatography-tandem mass spectrometry. J. Chromatogr. A.

[47] Drouet S., Garros L., Hano C., Tungmunnithum D., Renouard S., Hagège D., Maunit B., Lainé É. (2018). A Critical View of Different Botanical, Molecular, and Chemical Techniques Used in Authentication of Plant Materials for Cosmetic Applications. Cosmetics.

[48] Tungmunnithum D., Thongboonyou A., Pholboon A., Yangsabai A. (2018). Flavonoids and Other Phenolic Compounds from Medicinal Plants for Pharmaceutical and Medical Aspects: An Overview. Medicines.

[49] Chen H., Sun K., Yang Z., Guo X., Wei S. (2018). Identification of Antioxidant and Anti- α -amylase Components in Lotus (*Nelumbo nucifera*, Gaertn.) Seed Epicarp. Appl. Biochem. Biotechnol..

[50] Jiang X.L., Wang L., Wang E.J., Zhang G.L., Chen B., Wang M.K., Li F. (2018). Flavonoid glycosides and alkaloids from the embryos of *Nelumbo nucifera* seeds and their antioxidant activity. Fitoterapia.

[51] Wang M., Shi J., Wang L., Hu Y., Ye X., Liu D., Chen J. (2018). Inhibitory kinetics and mechanism of flavonoids from lotus (*Nelumbo nucifera* Gaertn.) leaf against pancreatic α-amylase. Int. J. Biol. Macromol..

[52] Liao L., Chen J., Liu L., Xiao A. (2018). Screening and binding analysis of flavonoids with alpha-amylase inhibitory activity from lotus leaf. J. Braz. Chem. Soc..

